# Expansion of the antimicrobial peptide repertoire in the invasive ladybird *Harmonia axyridis*

**DOI:** 10.1098/rspb.2012.2113

**Published:** 2013-01-07

**Authors:** Andreas Vilcinskas, Krishnendu Mukherjee, Heiko Vogel

**Affiliations:** 1 Institute of Phytopathology and Applied Zoology, Justus-Liebig University, Heinrich-Buff-Ring 26-32, 35392 Giessen, Germany; 2 Germany Department of Entomology, Max Planck Institute for Chemical Ecology, Beutenberg Campus, Hans-Knoell-Strasse 8, 07745 Jena, Germany

**Keywords:** invasion biology, innate immunity, gene family expansion, antimicrobial peptides, *Harmonia axyridis*

## Abstract

The harlequin ladybird beetle *Harmonia axyridis* has emerged as a model species in invasion biology because of its strong resistance against pathogens and remarkable capacity to outcompete native ladybirds. The invasive success of the species may reflect its well-adapted immune system, a hypothesis we tested by analysing the transcriptome and characterizing the immune gene repertoire of untreated beetles and those challenged with bacteria and fungi. We found that most *H. axyridis* immunity-related genes were similar in diversity to their counterparts in the reference beetle *Tribolium castaneum*, but there was an unprecedented expansion among genes encoding antimicrobial peptides and proteins (AMPs). We identified more than 50 putative AMPs belonging to seven different gene families, and many of the corresponding genes were shown by quantitative real-time RT–PCR to be induced in the immune-stimulated beetles. AMPs with the highest induction ratio in the challenged beetles were shown to demonstrate broad and potent activity against Gram-negative bacteria and entomopathogenic fungi. The invasive success of *H. axyridis* can therefore be attributed at least in part to the greater efficiency of its immune system, particularly the expansion of AMP gene families and their induction in response to pathogens.

## 1. Introduction

Invasive species have a negative impact on biodiversity and the economy, but it remains unclear why some species become successful invaders and others, even closely related ones, do not [1–3]. One property that may encourage invasive success is the ability to mount a strong humoral immune response, since this should facilitate the colonization of new habitats containing unfamiliar pathogens [4]. However, immune responses are costly in terms of resources and thus compete with other fitness traits that are essential for invasive success, such as fecundity [5,6].

The role of immunity in invasion biology has been investigated in the harlequin ladybird beetle *Harmonia axyridis* (also known as the multi-coloured Asian ladybird) because it is an established model invasive species [3]. *Harmonia axyridis* is native to central Asia but has been introduced into many regions to control aphids and other pests, and has now started displacing native ladybirds and threatening insect biodiversity in general [7]. The invasive success of *H. axyridis* has been investigated and recent studies have shown that it is better protected against intra-guild predation than native ladybird species [8]. *Harmonia axyridis* is only sporadically infected by parasitoids and the insects usually survive these attacks [3,7]. In accordance with this hypothesis, *H. axyridis* is also highly resistant to entomopathogenic nematodes and fungi that tend to kill native ladybirds [9]. For example, native species such as the two-spotted ladybird (*Adalia bipunctata*) and the seven-spotted ladybird (*Coccinella septempunctata*) are killed by moderate doses of the fungal pathogen *Beauveria bassiana*, whereas much higher doses are required to kill *H. axyridis* [10]. The components responsible for the robust innate immunity of *H. axyridis* were unknown until we recently discovered a strong constitutive antibacterial activity in the haemolymph of *H. axyridis*, which is absent in *A. bipunctata* and present at much lower levels in *C. septempunctata* [11]. The active compound was identified by mass spectrometry as harmonine, also known as (17*R*,9*Z*)-1,17-diaminooctadec-9-ene, a secondary metabolite with broad-spectrum antibacterial activity *in vitro*, including activity against human pathogens such as *Mycobacterium tuberculosis* and *Plasmodium falciparum* [11]. However, harmonine does not display *in vitro* activity against the fungal pathogen *B. bassiana* at the concentrations found in the haemolymph, suggesting other components of the immune system must confer fungal resistance.

Antimicrobial peptides (AMPs) are known to confer resistance to fungal pathogens [12–14], but little is known about the diversity and activity of these molecules in most insects, making it difficult to determine whether they play a role in invasive success. Forward genetics approaches, such as the analysis of insect transcriptomes, have resulted in the discovery of many immune-related genes and the corresponding networks and pathways involved in pathogen recognition, signal transduction and effector functions, including AMPs [15].

We therefore set out to identify candidate resistance genes in *H. axyridis* by comparing the transcriptomes of beetles injected with bacterial suspensions with those of untreated controls. We identified a number of induced genes encoding AMPs, including two strongly induced AMPs that were active against Gram-negative bacteria and entomopathogenic fungi. We also found a remarkable difference between *H. axyridis* and the model species *Tribolium castaneum* in the number of paralogues within several AMP gene families. This provides evidence for a two-layered innate immune system in *H. axyridis*, based on AMPs and harmonine, which may play a role in promoting the invasive success of this species.

## 2. Material and methods

### (a) Insect material and RNA isolation

*Harmonia axyridis* adults were collected in and around Giessen, Jena and Ober-Moerlen, Germany, for captive breeding. The beetles were reared in cages at 26°C and 60 per cent relative humidity with a 16-h photoperiod. Bean plants (*Phaseolus vulgaris*) infested with pea aphids (*Acyrthosiphon pisum*) were maintained as a food source as previously described [16].

We injected 5 µl of bacteria or yeast cells suspended in phosphate-buffered saline (PBS) into the haemocoel of adult beetles 24 h before dissection. The suspensions contained 10 mg ml^−1^ lyophilized *Escherichia coli, Micrococcus luteus* or *Saccharomyces cerevisiae*. Total RNA was obtained from adults and eggs (five individuals per sample) using Trizol reagent (Invitrogen) and was treated with DNase (Turbo DNase, Ambion) to eliminate contaminating genomic DNA. The RNA was purified using the RNeasy MinElute Cleanup Kit (Qiagen) and eluted in 20 μl of RNA Storage Solution (Ambion). RNA integrity was verified using an Agilent 2100 Bioanalyzer and RNA Nanochip (Agilent Technologies, Palo Alto, CA, USA), and RNA quantity was determined using a Nanodrop ND-1000 spectrophotometer. Equal amounts of total RNA from egg, beetle and challenged beetle samples were pooled to maximize the number of gene objects and reduce the number of unusable contigs during normalization and sequencing.

### (b) Normalization and construction of the cDNA library

Poly(A)^+^ RNA was prepared from the total RNA and used as the template for cDNA synthesis with N6-randomized primers. The cDNAs were then capped with 454 adapters A (5′-CCA TCT CAT CCC TGC GTG TCT CCG ACT CAG-3′) and B (5′-CTG AGA CTG CCA AGG CAC ACA GGG GAT AGG-3′) and amplified for 18 cycles using a proof-reading enzyme (Phusion DNA polymerase, Thermo Scientific). The optimal number of cycles for double-stranded cDNA synthesis was determined empirically by testing amplification products by electrophoresis to rule out signs of overcycling [17]. Normalization was achieved by one cycle of denaturation and reassociation, resulting in a mixture of single-stranded and double-stranded cDNAs. Reassociated double-stranded cDNA was separated from the normalized cDNA by passing the mixture over a hydroxyapatite column and amplifying the single cDNA strands for 11 cycles [17]. The resulting double-stranded cDNA pool was purified and concentrated using the DNA Clean and Concentrator Kit (Zymogen) and size fractionated on SizeSep 400 spun columns (GE Healthcare) with a cutoff of approximately 150 bp. A proportion of the double-stranded cDNA was cloned in vector pDNR-Lib (Clontech). Bacterial transformation, plasmid minipreparation, single-pass sequencing of cDNA library clones and sequence assembly were carried out as described [18]. Normalized cDNA material for Roche 454 sequencing was purified for direct sequencing without cloning.

### (c) Sequencing, assembly and annotation

The normalized cDNA library was used for 454 pyrosequencing on a Roche 454 FLX instrument and Sanger sequencing on an ABI 3730XL automatic DNA sequencer (PE Applied Biosystems). We sequenced 384 cloned cDNAs using the Sanger method to test the normalization efficiency and cDNA library quality. The pool of cDNAs from the normalization procedure was converted into a Roche 454 sequencing library according to the manufacturer's protocols, titrated and transferred to microtitre plates for processing at MWG Eurofins. The 454 sequence reads were preprocessed by masking poly(A) tails and removing SMART adapters using custom-written Perl scripts, trimmed for length and quality with standard settings and assembled using the CLC GENOMICS WORKBENCH v. 5.0.1 (CLC bio, Muehltal, Germany) with the following paramet­ers: nucleotide mismatch cost = 2; insertion = deletion costs = 2; length fraction = 0.4; similarity = 0.9; conflict among individual bases resolved by voting for the base with the highest frequency. Contigs shorter than 250 bp were removed from the final analysis. The total number of reads was 1 184 306 with an average read length of 296 bases. The data assembly comprised 48 483 contigs and 27 million bases including 21 454 contigs longer than 500 bases. The largest contig was 6702 bases in length, and the average contig size was 565 bases.

BLASTx and BLASTn homology searches and functional annotation using gene ontology (GO) terms (http://www.geneontology.org), InterPro terms (InterProScan, EBI), Enzyme Commission numbers and metabolic pathways in the Kyoto Encyclopedia of Genes and Genomes (KEGG) were carried out using the BLAST2GO software suite v. 2.4.1 [19]. A rough estimate of transcriptome coverage was determined by identifying all hits against the conserved KEGG pathway database and an estimate of genome coverage was achieved by identifying the complete ribosomal protein dataset compared with the full set from *T. castaneum*. Based on these findings, the estimated transcriptome coverage for moderately to highly expressed genes was near completion (e.g. 78 of 79 *T. castaneum* ribosomal proteins were identified). Nucleotide sequences were analysed in more detail using the commercial Lasergene software package and BioEdit freeware. Digital gene expression analysis was carried out using the CLC GENOMICS WORKBENCH by mapping each 454 read to the reference backbone sequence, which was then used to estimate relative expression levels (average contig coverage). In order to derive a non-redundant set of AMPs, we carefully analysed the different *H. axyridis* AMP sequences using a more conservative approach and excluded from the analysis any protein sequences differing by a single amino acid. We also designed primers specific for each AMP gene family and gene family member wherever possible, and used these to amplify *H. axyridis* AMP cDNAs and to verify them by standard Sanger sequencing. All of the AMPs used for our analysis could thus be verified by sequencing individual clones, eliminating the possibility of chimeric sequences deduced from potentially false contig assemblies. However, we cannot rule out the possibility that some of the AMP sequences represent (rare) allelic variants and are not necessarily different genes because environmental heterogeneity and pathogen diversity can maintain host genetic variation in immune function by favouring the spatio-temporal distribution of alternative genotypes [20]. On the other hand, this diversity of AMPs could not be found in any of the other extensive transcriptomic studies we and others have performed.

### (d) Analysis of AMP gene expression

We used quantitative real-time RT–PCR to compare the relative expression levels of a range of AMP genes belonging to different AMP gene families in control and immunized adult *H. axyridis* beetles and untreated eggs. Total RNA (500 ng) pooled from the tissues of 10 adults was reverse transcribed with a 3 : 1 mix of random and oligo-dT_20_ primers, and quantitative real-time PCR was carried out in optical 96-well plates on a Stratagene MX 3000P system using the Verso SYBR Green 2-Step QRT-PCR Kit Plus ROX Vial (Thermo Scientific) following the manufacturer's instructions. The specific amplification of transcripts was verified by dissociation curve analysis. All primers were designed using Primer3 (v. 0.4.0). Transcripts encoding eukaryotic initiation factor 4A and ribosomal protein RPS3 were used as references for normalization. Raw data and cycle threshold values (*C*_t_) were analysed with QBASE, using fold change of relative expression level in the immunized beetles compared with the sham-injected control beetles or control eggs, with lower transcript abundance (in the control beetles) set to 1.

### (e) Sequence alignment and phylogenetic reconstruction

Amino acid sequences were aligned using ClustalW v. 2 [21] or MAFFT [22] and visually inspected for regions of high-quality alignment. Phylogenetic reconstruction was carried out using Bayesian inference (MrBayes v. 3.1) or PhyML on the web-based phylogeny server (http://www.phylogeny.fr). For Bayesian analysis, the prior was set for the amino acid models to mix, thereby allowing model jumping between fixed-rate amino acid models. Markov chain Monte Carlo runs were carried out for 10 000 000 generations after which log-likelihood values showed that equilibrium had been reached after the first 5000 generations in all cases, and those data were discarded from each run and considered as ‘burnin’. Two runs were conducted for the dataset showing agreement in topology and likelihood scores. The maximum-likelihood and Bayesian tree topologies were in agreement, including their general subfamily relationships and node supports. The gene trees were visualized and optimized with the MEGA5 v. software package [23]. All of the deduced AMP protein sequences from *H. axyridis*, other Coleopteran and insect ESTs and NCBI sequences used in the phylogenetic analysis can be found in the electronic supplementary material, file S1.

### (f) Antimicrobial assays

Haxy_Col1 and Haxy_Col6 were dissolved in equal volumes of dimethylformamide (Sigma) to a final concentration of 50 mg ml^−1^ (Haxy_Col6) or 80 mg ml^−1^ (Haxy_Col1). Gram-positive *M. luteus* and Gram-negative *E. coli* D31 were maintained on LB agar plates (Carl Roth). Bacteria in their exponential growth phase were diluted in LB broth to OD_600_ = 0.02 and supplemented with the AMPs. At least three measurements of bacterial growth were performed. The optical density at 37°C was measured at 20 min intervals. After 24 h, the samples were serially diluted in PBS and plated on LB agar. The colony-forming units were counted after an overnight incubation at 37°C. The fungus *Metarhizium anisopliae* strain 43 (obtained from the Julius-Kühn-Institute, Darmstadt, Germany) was maintained on potato dextrose agar (Carl Roth) and conidia were isolated as described [24]. Approximately 16 000 conidia were inoculated in potato dextrose broth (Carl Roth) supplemented with the AMPs and incubated at 27°C. The germinating conidia were counted 22 h later under an optical microscope (Leica).

## 3. Results and discussion

### (a) Identification of *Harmonia axyridis* transcripts encoding antimicrobial peptides and other immune-related proteins

**Figure 1.**
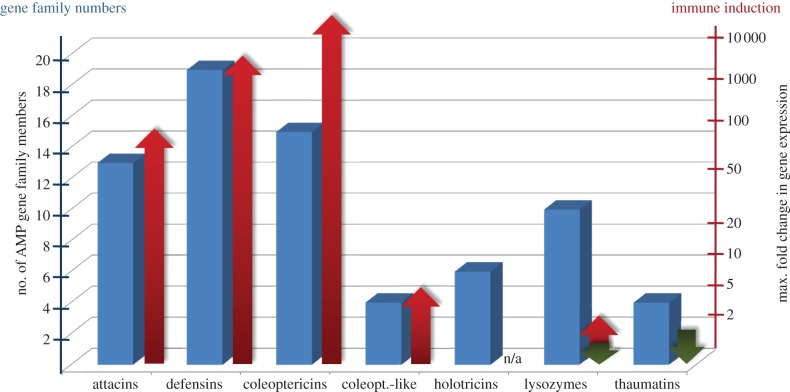
*Harmonia axyridis* AMP gene family member numbers and maximum immune-induction levels. Putative numbers of gene family members based on identified transcripts for each of the AMP gene families in *H. axyridis*. Differential gene expression in immune-induced *H. axyridis* adults compared with control beetles is depicted by coloured arrows (red = upregulation; green = downregulation). For normalization of transcript quantities, RPS3 and eIF4A were used.

We initiated a next-generation transcriptome sequencing program for *H. axyridis* to make up for the lack of publically available sequence data. Total RNA was isolated from untreated eggs and beetles reared under controlled conditions and from beetles challenged by injecting a bacterial and fungal suspension to induce immune-related genes. Equal amounts of RNA from each sample were pooled and sequenced using the Roche 454 FLX platform, and 1.1 million reads were assembled *de novo* to produce approximately 48 000 contigs. These were used as BLASTx and BLASTn search queries to identify homologues in other species, and functional annotation was carried out using the BLAST2GO software suite to match unique genes against GO terms, InterPro terms, EC numbers and KEGG metabolic pathways. A digital gene expression analysis was performed by mapping the individual 454 reads back to the reference backbone (contig sequences). The most highly expressed genes (top 200 contig sequences with highest read coverage) in the moderately normalized *H. axyridis* library encoded proteins involved in the mitochondrial respiratory chain and ATP synthesis (cytochrome c, vacuolar ATP synthase, electron transfer flavoprotein), general cellular homeostasis, ribosomal proteins and also many immune effector genes (see the electronic supplementary material, file S2). An extensive search for cDNAs encoding AMPs and lysozymes resulted in the identification of a remarkably large and diverse spectrum of AMPs, vastly exceeding the number found in other insect species (figure 1).

#### (i) Attacins

Ten genes encoding attacins were found in the *H. axyridis* transcriptome (figure 1), compared with three identified in the *T. castaneum* genome. Attacins were first discovered in the silk moth *Hyalophora cecropia* (family Attacidae) and are characterized by a molecular mass of approximately 20 kDa and a high content of glycine residues [25]. Attacins bind to bacterial lipopolysaccharides; therefore, basic attacin is more active against *E. coli* than the acidic form. Further, attacin reportedly blocks the translation of genes encoding outer membrane proteins when it binds to the bacterial cell surface [25]. Interestingly, allelic divergence and paralogous gene conversion has been reported among the attacin genes of *Drosophila melanogaster* [26]. Whether or not these mechanisms caused the diversification of attacins in *H. axyridis* will be addressed in future work.

#### (ii) Coleoptericins

**Figure 2.**
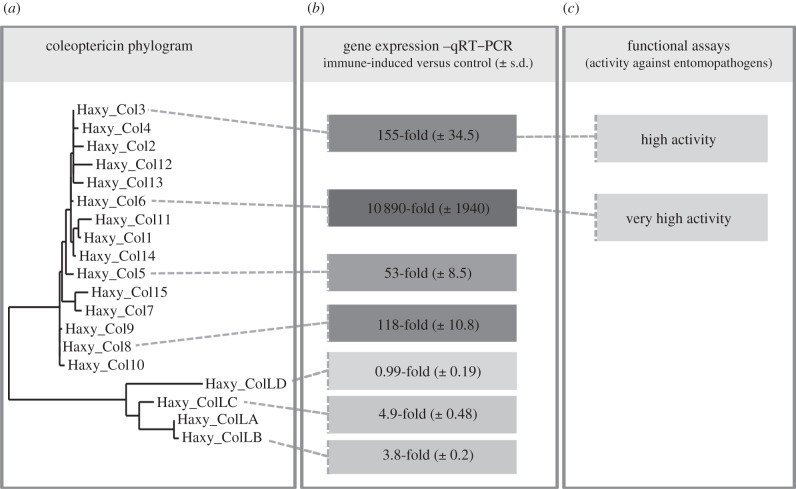
Gene phylogeny, gene expression and activity of *H. axyridis* coleoptericin proteins. (*a*) Maximum-likelihood phylogenetic tree of *H. axyridis* coleoptericin (Haxy_Col) and coleoptericin-like (Haxy_ColL) proteins. (*b*) Differential gene expression in *H. axyridis* immune-induced beetles compared with control beetles is depicted for a selected set of coleoptericins and coleoptericin-like genes. Values are shown as immune-induced versus control beetle fold changes with s.d. shown in brackets. Higher fold-change values are depicted in darker grey, whereas lower changes in gene expression are depicted in light colours. (*c*) Functional assay of two coleoptericin peptides displaying activity against entomopathogenic fungi.

Fifteen genes encoding coleoptericins and four encoding coleoptericin-like AMPs were identified in the *H. axyridis* transcriptome (figure 2), which is a significant expansion compared with the two coleoptericins encoded in the *T. castaneum* genome [27]. Coleoptericins are structurally similar to attacins and are rich in glycine and proline residues. The first coleoptericin was discovered in *Allomyrina dichotoma* larvae challenged with bacteria [28], and a further member of this protein family has been identified in the burying beetle *Nicrophorus vespilloides* [29]. The deduced amino acid sequences of all the *H. axyridis* coleoptericins include a signal peptide for extracellular localization and the conserved furin cleavage site, yielding a mature peptide approximately 75 amino acids in length (see the electronic supplementary material, figure S1). Although the four *H. axyridis* coleoptericin-like proteins also contain these conserved motifs, the predicted mature peptides are approximately 15 amino acids shorter than the coleoptericins identified thus far, resulting in a clear phylogenetic separation (figure 2). The activity of coleoptericin peptides against Gram-negative bacteria (including methicillin-resistant *Staphylococcus aureus*) is not mediated by the formation of membrane pores but by interference with cell division [28]. Interestingly, a recent study provides evidence that the weevil *Sitophilus zeamais* uses coleoptericins to maintain control over their endosymbiontic bacteria, although it is unclear whether *H. axyridis* uses a similar strategy [30].

#### (iii) Defensins

At least 19 genes encoding defensins were identified in the *H. axyridis* transcriptome (figure 1) compared with the four defensin and defensin-like AMPs identified in the *T. castaneum* genome using bioinformatics and experimental approaches [27,31]. Defensins are cationic AMPs found in plants, fungi and animals, which are characterized by a triple-stranded β-hairpin structure and six conserved disulphide-linked cysteine residues [32]. The phylogenetic analysis of insect defensins suggests there are three subfamilies in insects [33]. The *H. axyridis* defensin genes we identified are induced following a bacterial challenge (see the electronic supplementary material, figure S2), in agreement with similar experiments in other beetles [29,31]. Most insect defensins display antibacterial activity whereas some, such as gallerimycin from the greater wax moth *Galleria* m*ellonella*, act exclusively against mycelium-forming fungi [12].

#### (iv) Thaumatins

We identified four genes encoding thaumatins in the *H. axyridis* transcriptome, the same number identified in *T. castaneum* [31]. Thaumatins are frequently found in plants, and have also been identified in the nematode *Caenorhabditis elegans* [34] and in the pea aphid *A. pisum* [35]. These AMPs are larger and more complex than attacins and coleoptericins, with approximately 200 amino acid residues and multiple disulphide bridges. The potent antifungal activity of *T. castaneum* thaumatin was preserved when a recombinant version was tested *in vitro* [31].

#### (v) Lysozymes

We identified four chicken-type (C-type) and six invertebrate-type (I-type) lysozyme genes in the *H. axyridis* transcriptome, which is similar to or higher than the number found in other insects, depending on the species [36]. C-type lysozymes degrade peptidoglycans in the bacterial cell wall by hydrolysing the *β*(1–4) linkages between *N*-acetylglucosamine and *N*-acetylmuramic acid residues, hence their predominant activity against Gram-positive bacteria [32]. However, the C-type lysozyme from the greater wax moth *G. mellonella* also shows moderate activity against Gram-negative bacteria and fungi [37]. I-type lysozymes contain five or six disulphide bonds, explaining their thermostability and resistance against proteolytic degradation, but their biological function remains to be determined [36]. Phylogenetic analysis clustered the four C-type lysozymes from *H. axyridis* and the five from *T. castaneum*, but indicated that gene duplication events had occurred after speciation. Likewise, the six I-type lysozymes from *H. axyridis* and the four from *N. vespilloides* clustered together but gathered in species-dependent subclusters (see the electronic supplementary material, figure S3), which would indicate that the majority of I-type lysozymes evolved by gene duplication events preceding speciation.

### (b) AMP diversity in *Harmonia axyridis* and comparison to other immunity-related genes

It has been proposed that, depending on the population size and diversity, invasive species in new environments should go through a population bottleneck, reducing the levels of heterozygosity and potentially leading to inbreeding depression. However, a recent comparison of native and introduced *H. axyridis* populations showed that although the introduced populations experienced a population bottleneck of intermediate intensity, the resulting inbreeding depression was no worse than that suffered by native populations, perhaps because deleterious alleles were purged thus promoting high fitness even when inbred [38]. This would suggest that the limited genetic variation in introduced *H. axyridis* populations would increase the mean fitness relative to more genetically diverse native populations. This limited genetic variation should on the other hand also lead to reduced maximum numbers of alleles (including those derived from genes coding for AMPs) identified in the invasive *H. axyridis* population used for our studies. However, although this is true for almost all of the transcripts we analysed (including immune-related ones), the AMP-related transcripts violate this hypothesis.

The genome of the reference beetle *T. castaneum* appears to encode at least 16 AMPs and four lysozymes when experimental and bioinformatics data are combined [27,31]. In contrast, the immunity-related transcriptome of *H. axyridis* contains a slightly larger number of lysozyme genes but a vastly expanded repertoire of at least 50 AMP genes representing several different AMP families. It would be preferable to compare the immune gene repertoire of *H. axyridis* directly with those from non-invasive ladybirds such as *C. septempunctata* but there is insufficient published sequence data for such comparisons, making *T. castaneum* the best reference genome currently available. Although this may present drawbacks in terms of the taxonomic distance between the species and their different diets and lifestyles, the fact that most immunity-related genes are present in similar numbers in both species and only certain AMP families have expanded in *H. axyridis* suggests that the comparison is nevertheless valid. The diversity in both cases appears to have largely arisen by gene duplication after speciation. The remarkably dynamic evolution of AMPs in insects is likely to represent successful adaptations to a changing pathosphere [15], although there is no consensus on how such gene duplications are fixed and maintained in the genome [39].

We compared the expansion found in several of the AMP gene families to other immunity-related genes, such as pattern recognition receptor genes (GNBPs and PGRPs). This revealed that the number of paralogues was similar in *H. axyridis* to the reference beetle *T. castaneum* (five GNBPs and four PGRPs), suggesting that some AMP-encoding gene families are potentially more versatile than proteins that bind to microbial cell wall components. Our observation supports the hypothesis that genes encoding immunity-related effector molecules are more evolutionarily dynamic than genes encoding signalling or pathogen recognition proteins, reflecting their ability to adapt more rapidly [15].

### (c) Immune gene expression analysis

As well as showing the most remarkable phylogenetic expansion, the AMP genes were well-represented among the top 200 most strongly expressed genes in the *H. axyridis* transcriptome, calculated by mapping individual reads against the contigs generated by *de novo* assembly (see the electronic supplementary material, file S2). Indeed, the strongly expressed genes were dominated by three AMP families: the attacins, coleoptericins and defensins. When relative expression levels were presented as average coverage values (which combines the length of the contig with the number of mapped sequence reads), we found that five attacins, six coleoptericins and six defensins were among the transcripts with 150 or more read coverage, similar to strongly expressed housekeeping genes such as those encoding NADH dehydrogenases, cytochrome oxidases and ribosomal proteins.

A subset of the AMP and lysozyme genes was analysed in more detail by quantitative real-time RT–PCR to determine the induction ratio in challenged insects. Several of the coleoptericin-like and defensin family AMPs (and one lysozyme gene) showed moderate induction (2–10-fold), but certain coleoptericins (e.g. Haxy_Col6 and Haxy_Col1), attacins and defensins were induced several hundred to thousand-fold following bacterial challenge (figure 1), consistent with their ranking in the top 200 most strongly expressed *H. axyridis* genes. It is noteworthy that induction was not strictly AMP family-dependent, e.g. whereas some coleoptericins were strongly or moderately induced, others such as Haxy_ColLD were not induced at all under our experimental conditions. None of the thaumatins were induced in the challenged beetles and some were even downregulated, which is also consistent with the absence of any thaumatin genes in the top 200 ranking. Similarly, only one of the C-type lysozyme genes was moderately induced as noted above, while the rest were either unaffected or downregulated. However, this contrasts with data from other insects where at least one of the C-type lysozymes usually displays a strong immune induction [36].

We also tested the expression of AMPs and lysozymes in untreated *H. axyridis* eggs. All of the AMPs and all but one of the C-type lysozymes were expressed in the eggs at levels close to or below the detection threshold, whereas the control housekeeping transcripts (EIF-4a and RPS3) were expressed at similar levels in eggs and adults with and without induction. *Harmonia axyridis* eggs are known to possess a strong antibacterial activity [11] but our data suggest this is not conferred by the presence of AMPs.

### (d) Coleoptericin activity assays

**Figure 3.**
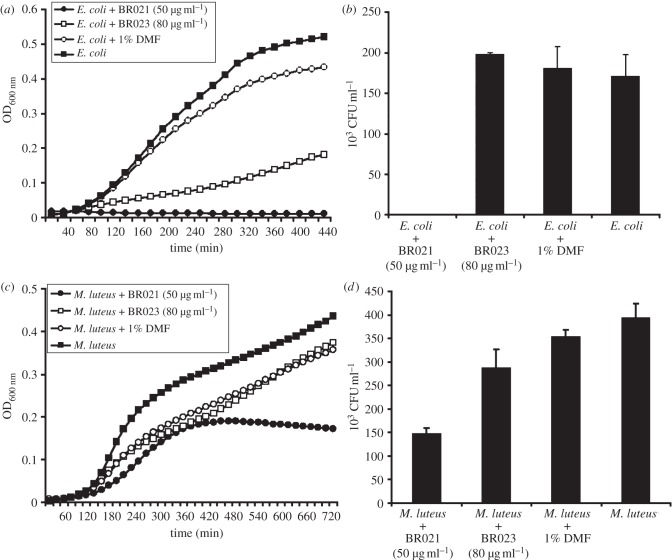
Activity of *H. axyridis* coleoptericins against bacteria. Growth of *E. coli* (*a*,*b*) and *M. luteus* (*c*,*d*) in the presence of coleoptericins (BR021 and BR023) from *H. axyridis*. Bacteria were cultured in LB broth supplemented with BR021 (50 μg ml^−1^) and BR023 (80 μg ml^−1^), and the optical density was measured every 20 min at 600 nm (*a*,*c*). The bacterial cultures after 24 h were serially diluted and grown on LB agar for the estimation of colony-forming units (*b*,*d*). Experiments were repeated three times with similar results.

**Figure 4.**
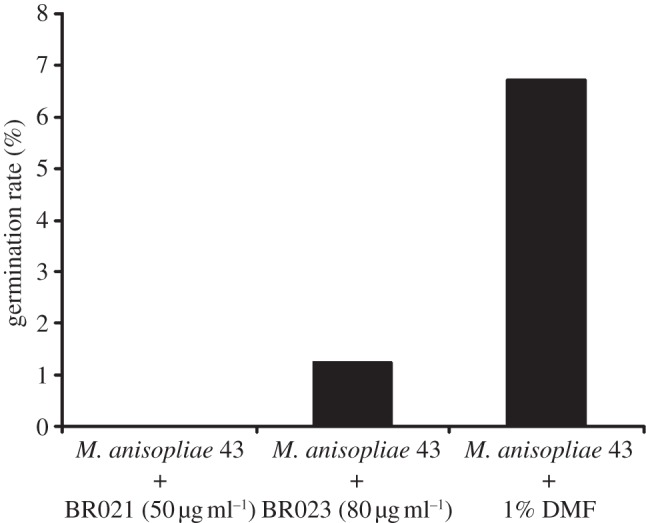
Activity of *H. axyridis* coleoptericins against entomopathogenic fungi. Germination rate of *M. anisopliae* conidia in the presence of coleoptericins (BR021 and BR023) from *H. axyridis*. The fungus was cultured in PDB supplemented with BR021 (50 μg ml^−1^) and BR023 (80 μg ml^−1^), and the germination rate was measured 22 h after inoculation. Experiments were repeated two times with similar results.

The strong induction of a subset of the *H. axyridis* AMP gene repertoire in response to bacterial challenge led us to investigate their activities against a spectrum of pathogens. We synthesized the predicted mature peptides encoded by the two most strongly expressed AMPs, which were both coleoptericins (Haxy_Col1 and Haxy_Col6). The mature peptides differed at eight amino acids (see the electronic supplementary material, file S3 and figure S4). The synthetic peptides were tested against a Gram-negative bacterium (*E. coli*), a Gram-positive bacterium (*M. luteus*) and an entomopathogenic fungus (*M. anisopliae*). Haxy_Col1 and Haxy_Col6 inhibited the growth of Gram-negative bacteria strongly, with Haxy_Col6 showing the stronger activity of the two AMPs (figure 3*a,b*). However, they showed only moderate (Haxy_Col6) to low (Haxy_Col1) activity against Gram-positive bacteria (figure 3*c*,*d*). Remarkably, the same AMPs achieved strong (Haxy_Col1) to complete (Haxy_Col6) inhibition of the germination of the entomopathogenic fungus *M. anisopliae* (figure 4).

**Figure 5.**
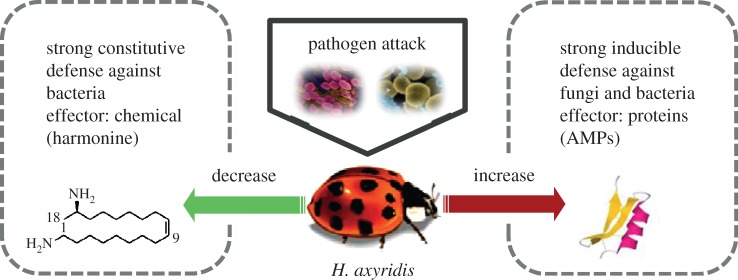
The two-layered immune defence system of *H. axyridis*. The innate immune defence system of *H. axyridis* is based on a harmonine-based constitutive chemical defence effective against bacteria and an inducible defence based on a highly diverse set of antimicrobial peptides effective against fungi and bacteria.

A coleoptericin has recently been shown to play a central role in host–symbiont interactions in the weevil *S. zeamais* [30]. The vertically transmitted endosymbionts are prevented from spreading and invading non-bacteriome tissues through the action of a specific AMP, coleoptericin-A, which selectively targets endosymbionts within the bacteriome and regulates their growth by inhibiting cell division. RNAi-based silencing of the coleoptericin gene prevented growth regulation and allowed the endosymbionts to escape and spread into insect tissues [30]. This study, among others, shows that AMPs can have effects other than the direct killing of bacterial and fungal pathogens. Few studies have tested individual AMPs against a broad spectrum of microbial species and strains. However, the growing cumulative evidence from reports on the sometimes highly variable activities of AMPs would support the idea that a highly diverse set of such antimicrobial effector proteins should allow the innate immune system to overcome a broad spectrum of emerging pathogens [15] that invasive species encounter in newly colonized habitats. The constitutive synthesis of these AMPs would require the allocation of resources from other traits relevant for invasive success, such as fecundity, the ability to cope with intra-guild predators or to adapt to different diets. Therefore, we postulate that the harmonine-based constitutive antimicrobial activity [11] is associated with a smaller fitness penalty and adds to a two-layered defence system that combines constitutive chemical defence with inducible AMP-based immune responses (figure 5). The proposed two-layered defence system is in line with a recent study using the beetle *Tenebrio molitor*, which implies that induced AMPs help to protect the insect host against bacteria that persist despite constitutive immune responses, rather than to clear microbial infections [40].

## 4. Conclusions

The invasive ladybird *H. axyridis* has a two-layered innate immune system, combining a constitutive chemical defence strategy based on the secondary metabolite harmonine (which is effective against a range of bacteria) and a broad range of inducible AMPs and defence enzymes (which are effective against both bacteria and entomopathogenic fungi; figure 5). The invasive success of *H. axyridis* may reflect both the robustness and flexibility of its antimicrobial defence that combines constitutive with inducible components. Particularly, the unprecedented diversity of its AMP repertoire which appears to result from multiple gene duplication and divergence events after speciation may contribute to the ability of *H. axyridis* to successfully fight pathogens both in its native range and in newly colonized habitats.
